# Potential Therapeutic Benefits of Metformin Alone and in Combination with Sitagliptin in the Management of Type 2 Diabetes Patients with COVID-19

**DOI:** 10.3390/ph15111361

**Published:** 2022-11-07

**Authors:** Hayder M. Al-Kuraishy, Ali I. Al-Gareeb, Sarah M. Albogami, Sabatier Jean-Marc, Eman Hassan Nadwa, Amin A. Hafiz, Walaa A. Negm, Marwa Kamal, Mohammed Al-Jouboury, Engy Elekhnawy, Gaber El-Saber Batiha, Michel De Waard

**Affiliations:** 1 Department of Clinical Pharmacology and Medicine, College of Medicine, AL-Mustansiriyia University, Baghdad 14132, Iraq; 2 Department of Biotechnology, College of Science, Taif University, Taif 21944, Saudi Arabia; 3 Aix-Marseille Université, Institut de Neurophysiopathologie (INP), CNRS UMR 7051, Faculté des sciences médi-cales et paramédicales, 27 Bd Jean Moulin, 13005 Marseille, France; 4 Department of Pharmacology and Therapeutics, College of Medicine, Jouf University, Sakaka 72345, Saudi Arabia; 5 Department of Medical Pharmacology, Faculty of Medicine, Cairo University, Cairo 12613, Egypt; 6 Department of Clinical Nutrition, Faculty of Applied Medical Sciences, Umm Al-Qura University, Mecca 24382, Saudi Arabia; 7 Department of Pharmacognosy, Faculty of Pharmacy, Tanta University, Tanta 31527, Egypt; 8 Clinical Pharmacy Department, Faculty of Pharmacy, Fayoum University, Fayoum 63514, Egypt; 9 Department of Community Medicine and Statistics, College of Medicine, Baghdad University, Baghdad 10071, Iraq; 10 Pharmaceutical Microbiology Department, Faculty of Pharmacy, Tanta University, Tanta 31527, Egypt; 11 Department of Pharmacology and Therapeutics, Faculty of Veterinary Medicine, Damanhour University, Damanhour 22511, Egypt; 12 Smartox Biotechnology, 6 Rue des Platanes, 38120 Saint-Egrève, France; 13 L’institut du thorax, INSERM, CNRS, Université de Nantes, 44007 Nantes, France; 14 LabEx «Ion Channels, Science & Therapeutics», Université de Nice Sophia-Antipolis, 06560 Valbonne, France

**Keywords:** cytokines, diabetes mellitus, metformin, SARS-CoV-2, sitagliptin

## Abstract

Type 2 diabetes mellitus (T2DM) is a potential risk factor for the development of COVID-19 and is associated with higher severity and mortality rates. T2DM patients are commonly treated with metformin monotherapy or metformin plus sitagliptin. In the present case-control, single-center cohort study, a total number of 112 T2DM patients suffering from COVID-19 and aged 44–62 years old were compared with 78 T2DM patients without COVID-19 and aged 42–56 years old. Both the patient group and the control group were allocated into four groups. Group A: T2DM patients with COVID-19 on metformin treatments plus standard therapy (*n* = 60); group B: T2DM patients with COVID-19 on metformin plus sitagliptin plus standard therapy (*n* = 52); group C: T2DM patients without COVID-19 on metformin treatments (*n* = 40); and group D: T2DM patients without COVID-19 on metformin plus sitagliptin (*n* = 38). The investigation duration was 2–3 weeks. Anthropometric measurements, serological and biochemical investigations, pulmonary radiological findings, and clinical outcomes were evaluated. Only 101 T2DM patients with COVID-19 continued the study, 71 (70.29%) with mild-moderate COVID-19 and 30 (29.7%) with severe COVID-19 were compared with 78 T2DM patients as a control. Inflammatory biomarkers (C reactive protein, ferritin, and procalcitonin), a lung injury biomarker (lactate dehydrogenase), and a coagulopathy biomarker (D-dimer) were elevated in severe COVID-19 patients compared with mild-moderate COVID-19 (*p* < 0.05) and T2DM patients (*p* < 0.05). However, metformin plus sitagliptin was more effective than metformin monotherapy in T2DM patients with COVID-19, as evidenced by the mitigation of oxidative stress, CT scan score, and clinical outcomes. The present study confirmed the protective effects of this combination against the development of COVID-19 severity, as most T2DM COVID-19 patients develop mild-moderate forms. Herein, the combination of metformin and sitagliptin may lead to more beneficial effects than metformin monotherapy.

## 1. Introduction

In 2019, many atypical respiratory diseases were recognized in Wuhan, China, known as Wuhan pneumonia. This disease was rapidly spread to other areas [1]. It was found that a new virus was responsible for this disease, named novel coronavirus 2019 (nCoV-2019) or humane coronavirus 2019 (HCoV-19), which was renamed severe acute respiratory syndrome type 2 (SARS-CoV-2) [2]. 

SARS-CoV-2 attaches to angiotensin-converting enzyme 2 (ACE2) receptors on the different cell surfaces [3]. The interaction between SARS-CoV-2 and ACE2 induces considerable downregulation of this receptor. ACE2 is involved in the regulation of the renin-angiotensin system (RAS). It metabolizes and converts pro-inflammatory and vasoconstrictor angiotensin II (AngII) to anti-inflammatory and vasodilator Ang1-7. Downregulation of ACE2 induces an increase in the circulating AngII with a reduction of Ang1-7. These changes lead to the augmentation release of pro-inflammatory cytokines and tissue injury. Furthermore, SARS-CoV-2 can attach to dipeptidyl peptidase 4 (DPP4), but somewhat to a lesser extent than ACE2 [4].

Risk factors that could lead to the emergence of COVID-19 are more related to individual factors, such as age, gender, and co-morbidities. Epidemiological studies indicated that older age groups over 60 years are more affected and vulnerable to COVID-19. It had been shown that 95% of COVID-19 patients were elderly, and 82% had one or more co-morbidities [4]. COVID-19 patients could be asymptomatic or present with mild flu-like illness in most of the cases (85%). However, 10–15% presented with moderate symptoms, including fever, headache, sweating, sore throat, anosmia, myalgia, dry cough, and dyspnea [4]. Severe cases, which account for about 5% of the COVID-19 patients, may develop severe symptoms, such as dyspnea, tachypnea, severe fever, and signs of hypoxemia due to the development of acute lung injury (ALI) and acute respiratory distress syndrome (ARDS), which developed in 20–40% of hospitalized COVID-19 patients [4]. The severe cases may progress to a critical state that needs intensive care unit (ICU) admission with assistance and/or mechanical ventilation. Most of the COVID-19 patients are recovered with low mortality without complications. Though they were hospitalized, severely and critically ill COVID-19 patients in the ICU had a higher mortality, reaching up to 50%, and most of the recovered patients may develop long-term complications called post-COVID 19 [5]. The main causes of increased mortality in COVID-19 patients are the development of cytokine storm and linked complications, such as ARDS, respiratory failure, multi-organ failure (MOF), and shock. 

COVID-19 can affect the pathogenesis and complications of T2DM. A high prevalence of T2DM is seen in COVID-19 patients, estimating the degree of severity and mortality [5]. Higher expression of ACE2 in T2DM may increase the risk of infection with COVID-19 and induced acute pancreatic injury (API) with the development of new-onset DM [6]. This pathologic mechanism may exacerbate underlying relative insulin deficiency. High pro-inflammatory cytokines and exaggerated immune response may induce new or exacerbate underlying insulin resistance (IR). Thus, API and IR may cause new-onset DM in COVID-19 with the development of hyperglycemia and blood glucose variability [6]. 

DPP4 plays a vital part in glucose metabolism by degradation and metabolism of incretins, such as glucose-dependent insulinotropic polypeptide (GIP) and glucagon-like peptide 1(GLP-1) [7]. Incretins increase insulin secretion and inhibit glucagon release with a subsequent reduction of blood glucose levels [7]. Thus, overexpression of DPP4 in patients suffering from diabetes mellitus is linked with hyperglycemia development [8]. DPP4 also has non-enzymatic activity; it activates T cell activity by producing co-stimulatory signals bound to adenosine deaminase [9]. Therefore, DPP4 could be a probable link between COVID-19 and DM, which may clarify the susceptibility of diabetes patients to the effect of COVID-19. 

Unfortunately, COVID-19 patients may suffer cardiovascular (CVS) complications, including arrhythmias, acute heart failure, and acute myocardial injury [10]. Moreover, they may suffer neurological manifestations, such as dizziness, headache, anosmia, muscle weakness, stroke, ataxia, seizure, visual impairment, neuropathy, movement disorders, delirium, and other neuropsychiatric disorders [11]. This is in addition to other manifestations, such as acute kidney injury [12] and GIT symptoms [13]. 

Different agents were suggested to be effective in COVID-19 depending on previous use in the SARS-CoV epidemic in 2003 or experimental and in silico studies. Some of these proposed agents entered clinical trials, such as colchicine, but their benefits in a clinical setting were not evaluated thoroughly [14]. Thus, there is no single drug to be efficient entirely against SARS-CoV-2 infection, and the utilized therapeutic agents in COVID-19 management depend on previous experiences [15].

Metformin is an antidiabetic drug belonging to the biguanide class with anti-hyperglycaemic activity. Metformin has been regarded as the first therapy to manage T2DM, mainly in overweight and obese patients [7]. Metformin improves peripheral insulin sensitivity through the increase in glucose uptake and oxidation through activated ions of adenosine monophosphate protein kinase (AMPK). Furthermore, metformin inhibits liver glycogenolysis and gluconeogenesis [7]. Metformin does not enhance insulin release from pancreatic β cells; thus, it does not cause hypoglycaemia.

Sitagliptin is an antidiabetic drug from the DPP4 inhibitors family, it acts through the inhibition of DPP4 leading to an increase in incretins with subsequent stimulation of insulin and inhibition of glucagon. Sitagliptin induces glucose-dependent insulin release, so it rarely causes hypoglycaemia. Both sitagliptin and metformin induce weight loss via the suppression of the feeding center and induction of taste aversion [8]. A combination of sitagliptin and metformin was permitted for the management of T2DM [16].

Therefore, the aim of the present study was to evaluate the influence of metformin and sitagliptin on inflammatory and cardiometabolic problems in T2DM patients suffering from COVID-19.

## 2. Results

**Figure 1:**
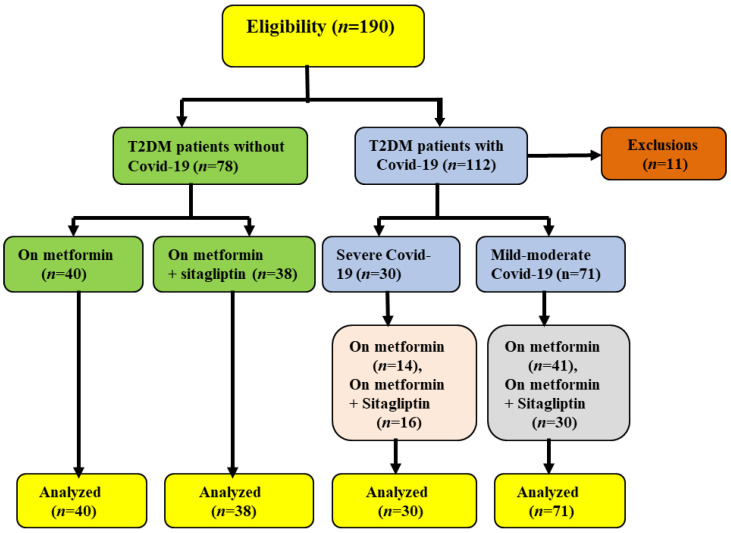
Consort-flow diagram of the current study.

As shown in Figure 1, only 101 T2DM patients with COVID-19 continued the study, 71 (70.29%) with mild-moderate and 30 (29.7%) with severe COVID-19 compared with 78 T2DM patients as a control. Table S1 provides information about these patients’ demographics. In addition, the clinical presentations of COVID-19 cases at the time of admission are shown in Table S2. 

### 2.1. Anthropometric and Biochemical Variables at Admission Time

#### 2.1.1. Effects of Metformin

Regarding the potential effect of metformin, 40 (42.10%) of the T2DM patients, 41 (43.15%) of the T2DM patients with mild-moderate COVID-19, and 14 (15.15%) of the T2DM patients with severe COVID-19 were on metformin monotherapy for about 3–5 years. In the present study, 55 (57.89%) of the T2DM patients developed COVID-19, 41 (74.54%) developed mild-moderate COVID-19, and only 14 (25.45%) developed severe cases.

In the present study, 55 (57.89%) of the T2DM patients developed COVID-19, of them 41 (74.54%) developed mild-moderate COVID-19, and only 14 (25.45%) developed severe COVID-19.

The BMI was higher in the T2DM patients with severe COVID-19 than in the T2DM patients with mild-moderate cases (*p* = 0.04) and T2DM patients (*p* = 0.03). SBP was not significant among groups. However, DBP, MAP, and PP were lower in T2DM patients with mild-moderate COVID-19 compared to T2DM patients (*p* = 0.0001). 

Regarding glucose indices, FBG was higher in mild-moderate and severe COVID-19 relative to T2DM patients (*p* = 0.0001). FBG was also higher in severe than in mild-moderate COVID-19 (*p* = 0.0001) cases. HbA1c was not altered significantly among groups (*p* = 0.59). Interestingly, FSI and HOMA2-IR were higher in severe COVID-19 patients than in mild-moderate COVID-19 and T2DM patients (*p* = 0.006). However, these variables did not significantly differ between mild-moderate COVID-19 and T2DM patients (*p* > 0.05). Furthermore, the pancreatic β-cell function was markedly reduced in severe COVID-19 patients compared with T2DM patients (*p* = 0.0001) and mild-moderate COVID-19 patients (*p* = 0.03). Likewise, the pancreatic β-cell function was significantly reduced in mild-moderate patients relative to T2DM patients (*p* = 0.0001). Insulin sensitivity (IS) was significantly reduced in severe COVID-19 patients compared with T2DM patients (*p* = 0.0001) and mild-moderate COVID-19 patients (*p* = 0.0001). Nonetheless, IS was not significantly reduced in mild-moderate COVID-19 patients compared to T2DM patients (*p* > 0.05).

Concerning lipid profile and cardiometabolic indices, TC, HDL, LDL, and VLDL serum levels were reduced in severe COVID-19 patients compared with T2DM patients (*p* < 0.05) and mild-moderate COVID-19 patients (*p* < 0.05). Although, the TG serum level was increased in severe COVID-19 patients compared with T2DM patients (*p* < 0.05) and mild-moderate COVID-19 patients (*p* < 0.05). This difference was not seen in mild-moderate COVID-19 patients compared to T2DM patients (*p* > 0.05). In the same manner, the non-HDL serum level was reduced in severe COVID-19 patients compared with T2DM patients only (*p* = 0.0001). In addition, AC and CVRI were higher in severe COVID-19 patients (*p* < 0.01) compared with T2DM patients, whereas AC was not significantly different compared with mild-moderate COVID-19 patients (*p* > 0.05).

CRR was not significant among groups (*p* = 0.06). Both AI and AC values were higher in mild-moderate COVID-19 patients as compared with T2DM (*p* = 0.0001). Moreover, SaO2% was low in severe COVID-19 patients compared with mild-moderate COVID-19 and T2DM patients (*p* = 0.0001). Nevertheless, SaO2% was not substantially different in mild-moderate COVID-19 relative to T2DM patients (*p* > 0.05). Further, pulmonary CT scan findings were higher in severe COVID-19 compared with mild-moderate COVID-19 (*p* = 0.0001). Of note, WBC and neutrophils:lymphocytes ratio (NLR) was more elevated in severe COVID-19 patients compared with mild-moderate COVID-19 (*p* = 0.0001) and T2DM patients (*p* = 0.0001). However, neutrophil counts and NLR were insignificant regarding mild-moderate COVID-19 compared to T2DM patients (*p* > 0.05). Of interest, inflammatory biomarkers (CRP, ferritin, and procalcitonin), a lung injury biomarker (LDH), and a coagulopathy biomarker (D-dimer) were elevated in severe COVID-19 patients compared with mild-moderate COVID-19 (*p* < 0.05) and T2DM patients (*p* = 0.0001), as shown in Table 1.

#### 2.1.2. Effects of Metformin plus Sitagliptin

Concerning the potential effect of metformin and sitagliptin, 38 (45.23%) of the T2DM patients, 30 (35.71%) of the T2DM patients with mild-moderate COVID-19, and 16 (19.04%) of the T2DM patients with severe COVID-19 were on metformin plus sitagliptin for about 3–5 years. In the present study, 46 (54.76%) T2DM patients developed COVID-19. Of them, 30 (65.21%) developed mild-moderate COVID-19, and only 16 (34.78%) developed severe COVID-19. In comparison with metformin monotherapy, there was no significant difference in the development of severe COVID-19 (difference = 9.33%, *p* = 0.30). Regarding the effect of metformin and sitagliptin on the anthropometric variables, this combination produced an insignificant effect on the BMI among groups (*p* > 0.05). SBP was low in T2DM patients with mild-moderate and severe COVID-19, but it was only significantly reduced in mild-moderate COVID-19 compared to T2DM patients (*p* = 0.03). DBP and MAP were low in mild-moderate and severe COVID-19 compared to T2DM patients (*p* = 0.0001). PP was low in severe COVID-19 compared to mild-moderate COVID-19 and T2DM patients (*p* = 0.0001). 

Concerning the effect of metformin plus sitagliptin on the glycemic indices, FBG was higher in both mild-moderate and severe COVID-19 compared to T2DM patients (*p* = 0.0001). HbA1c and FSI were not significantly different among the groups (*p* = 0.40) and (*p* = 0.09), respectively. HOMA2-IR was higher in severe COVID-19 compared to T2DM patients (*p* = 0.04), but it insignificantly differed from mild-moderate COVID-19. Pancreatic-β cell function was also low in both mild-moderate and severe COVID-19 compared to T2DM patients (*p* = 0.0001), but it did not significantly differ between patients with mild-moderate and severe COVID-19. IS was reduced in severe and mild-moderate COVID-19 patients compared to T2DM patients (*p* = 0.0001).

Regarding lipid profile and cardiometabolic indices, TC, HDL, non-HDL LDL, and VLDL were reduced in both mild-moderate and severe COVID-19 patients compared to T2DM patients (*p* < 0.05). Furthermore, TC and HDL were not significantly different between severe and mild-moderate COVID-19 patients (*p* > 0.05). However, the TG serum level was higher in both mild-moderate and severe COVID-19 patients compared to T2DM patients (*p* = 0.0001). AI was more elevated in severe COVID-19 patients compared to mild-moderate COVID-19 patients and T2DM patients (*p* = 0.0001). AC and CRR were not differed significantly among groups (*p* > 0.05), whereas CVRI was higher in severe COVID-19 patients compared to T2DM patients (*p* = 0.0008).

Furthermore, SaO2% was low in severe COVID-19 patients compared with mild-moderate COVID-19 and T2DM patients (*p* = 0.0001). Nonetheless, SaO2% was not significantly different in mild-moderate COVID-19 compared to T2DM patients (*p* > 0.05). Further, pulmonary CT scan findings were higher in severe COVID-19 patients compared with mild-moderate COVID-19 (*p* = 0.0001). The WBC and neutrophils: lymphocytes ratio (NLR) was more elevated in severe COVID-19 patients compared with T2DM patients (*p* = 0.0001); though, the neutrophil and lymphocyte counts and NLR were not different in severe COVID-19 compared to mild-moderate COVID-19 patients (*p* > 0.05). In addition, CRP, ferritin, procalcitonin, LDH, and D-dimer were higher in severe COVID-19 patients compared with mild-moderate COVID-19 and T2DM patients (*p* = 0.0001), as shown in Table 2.

#### 2.1.3. Effects of Metformin versus Metformin plus Sitagliptin

The effects of metformin and metformin plus sitagliptin were comparable. T2DM patients with mild-moderate COVID-19 on either metformin or metformin plus sitagliptin revealed no significant differences in most of the anthropometric and biochemical variables except for FBG, pancreatic β-cell function, and D-dimer, which were higher in T2DM patients with mild-moderate COVID-19 on metformin plus sitagliptin (*p* < 0.05). LDL serum levels were low in T2DM patients with mild-moderate COVID-19 on metformin plus sitagliptin compared to metformin monotherapy (*p* = 0.001).

Similarly, T2DM patients with severe COVID-19 on either metformin or metformin plus sitagliptin showed no significant differences on most of the anthropometric and biochemical variables except MAP, FBG, TG, AI, pancreatic β-cell function, and IS, which were better in severe COVID-19 patients on metformin plus sitagliptin relative to metformin monotherapy (*p* < 0.05). Interestingly, the lung CT scan score was lower in severe COVID-19 patients on metformin and sitagliptin than metformin monotherapy (*p* = 0.001), as shown in Table 3.

### 2.2. Oxidative Stress at Admission Time

#### 2.2.1. Impacts of Metformin on Oxidative Stress

**Figure 2:**
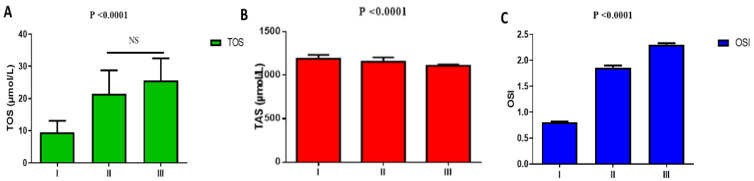
Metformin effects on oxidative stress in T2DM patients with COVID-19: I: T2DM patients on metformin, II: T2DM with mild-moderate COVID-19 on metformin, III: T2DM patients with severe COVID-19 on metformin. (**A**): TOS: total oxidant status; (**B**): TAS: total antioxidant status; (**C**): OSI: oxidative stress index. NS: not significant.

Regarding oxidative stress in T2DM with or without COVID-19, TOS was low in T2DM patients on metformin treatment (9.53 ± 3.55 µmol/L) as compared with T2DM patients on metformin treatment with mild-moderate COVID-19 (21.52 ± 7.22 µmol/L) and T2DM patients with severe COVID-19 (25.57 ± 6.92 µmol/L), (*p* = 0.0001). Moreover, TOS was not significantly different regarding mild-moderate COVID-19 as compared with severe COVID-19 patients (*p* > 0.05) (Figure 2A). Furthermore, TAS was higher in T2DM patients on metformin treatment (1190.42 ± 44.08 µmol/L) as compared with T2DM patients on metformin treatment with mild-moderate COVID-19 (1156.62 ± 49.09 µmol/L) and T2DM patients with severe COVID-19 (1110.06 ± 12.62 µmol/L), (*p* = 0.0001). TAS was more reduced in T2DM patients with severe relative to mild-moderate COVID-19 (*p* = 0.0001) (Figure 2B). In this state, OSI was low in T2DM patients on metformin treatment (0.80 ± 0.02) as compared with T2DM patients on metformin treatment with mild-moderate COVID-19 (1.86 ± 0.04) and T2DM patients with severe COVID-19 (2.30 ± 0.03L), (*p* = 0.001). However, TOS was low in mild-moderate COVID-19 relative to severe COVID-19 patients (*p* = 0.03) (Figure 2C). 

#### 2.2.2. Effect of Metformin plus Sitagliptin 

**Figure 3:**
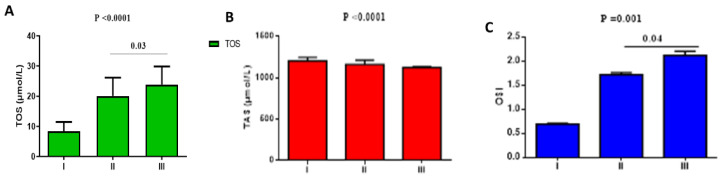
Effects of metformin plus sitagliptin treatment on the oxidative stress in T2DM patients with COVID-19: I: T2DM patients on metformin plus sitagliptin, II: T2DM patients with mild-moderate COVID-19 on metformin plus sitagliptin, III: T2DM patients with severe COVID-19 on metformin plus sitagliptin. (**A**): TOS: total oxidant status; (**B**): TAS: total antioxidant status; (**C**): OSI: oxidative stress index.

Concerning oxidative stress in T2DM with or without COVID-19, TOS was low in T2DM patients on metformin plus sitagliptin treatment (8.41 ± 3.08 µmol/L) as compared with T2DM patients on metformin plus sitagliptin treatment with mild-moderate COVID-19 (20.09 ± 6.08 µmol/L) and T2DM patients with severe COVID-19 (23.88 ± 6.01 µmol/L), (*p* = 0.0001). TOS also significantly differed regarding mild-moderate relative to severe COVID-19 patients (*p* = 0.03) (Figure 3A). Furthermore, TAS was higher in T2DM patients on metformin plus sitagliptin treatment (1204.32 ± 42.08 µmol/L) as compared with T2DM patients on metformin plus sitagliptin treatment with mild-moderate COVID-19 (1162 ± 50.22 µmol/L) and T2DM patients with severe COVID-19 (1123.06 ± 11.91µmol/L), (*p* = 0.0001). TAS was more reduced in T2DM patients with severe COVID-19 relative to mild-moderate COVID-19 (*p* = 0.0001) (Figure 3B). In this state, OSI was low in T2DM patients on metformin plus sitagliptin treatment (0.69 ± 0.02) as compared with T2DM patients on metformin plus sitagliptin treatment with mild-moderate COVID-19 (1.72 ± 0.04) and T2DM patients with severe COVID-19 (2.12 ± 0.09), (*p* = 0.001). However, OSI was low in mild-moderate COVID-19 as compared with severe COVID-19 patients (*p* = 0.04) (Figure 3C).

#### 2.2.3. Effect of Metformin versus Metformin plus Sitagliptin 

**Figure 4:**
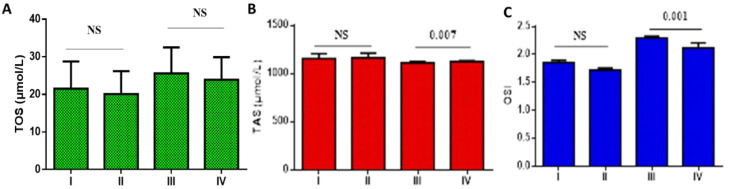
Effects of metformin monotherapy versus metformin plus sitagliptin treatment on the oxidative stress in T2DM patients with COVID-19: I: T2DM patients on metformin with mild-moderate COVID-19, II: T2DM patients on metformin plus sitagliptin with mild-moderate COVID-19, III: T2DM patients with severe COVID-19 on metformin monotherapy, IV: T2DM patients with severe COVID-19 on metformin plus sitagliptin. (**A**): TOS: total oxidant status; (**B**): TAS: total antioxidant status; (**C**): OSI: oxidative stress index. NS: not significant.

Concerning oxidative stress in T2DM with mild-moderate and severe COVID-19 on metformin monotherapy and metformin plus sitagliptin, TOS did not significantly differ in T2DM patients with mild-moderate COVID-19 on metformin monotherapy (21.52 ± 7.22 µmol/L) relative to T2DM patients with mild-moderate COVID-19 on metformin plus sitagliptin treatment (20.09 ± 6.08 µmol/L), (*p* > 0.05). Furthermore, TOS did not significantly differ regarding mild-moderate COVID-19 as compared with severe COVID-19 patients on metformin monotherapy or metformin plus sitagliptin treatment (*p* > 0.05) (Figure 4A). Furthermore, TAS did not significantly differ in T2DM patients with mild-moderate COVID-19 on metformin monotherapy (1156.62 ± 49.09 µmol/L) as compared with T2DM patients with mild-moderate COVID-19 on metformin plus sitagliptin treatment (1162 ± 50.22 µmol/L), (*p* > 0.05). TAS significantly differed regarding mild-moderate COVID-19 as compared with severe COVID-19 patients on metformin monotherapy or metformin plus sitagliptin treatment (*p* = 0.007) (Figure 4B). In this state, OSI was not significantly different regarding metformin monotherapy (1.86 ± 0.04) as compared to metformin plus sitagliptin treatment (1.72 ± 0.04) in T2DM patients with mild-moderate COVID-19 (*p* > 0.05). However, OSI was low in mild-moderate COVID-19 on metformin plus sitagliptin treatment (2.12 ± 0.09) relative to severe COVID-19 patients on metformin monotherapy (*p* = 0.001) (Figure 4C). 

### 2.3. Gender Assessment at Admission Time

Throughout the 101 T2DM patients with COVID-19, the number of recruited men was 79 (78.43%) compared to 22 (21.56%) recruited women (*p* = 0.001). A total of 45 (56.96%) men with COVID-19 were on metformin monotherapy compared to 34 (43.03%) on metformin plus sitagliptin (*p* = 0.38). A total of 11 (24.44%) men with COVID-19 were on metformin monotherapy and developed severe COVID-19 compared to 34 (75.56%) that developed mild-moderate COVID-19 (*p* = 0.03). In men with COVID-19 on metformin plus sitagliptin, 14 (41.17%) developed severe COVID-19 compared to 20 (58.82%) that developed mild-moderate COVID-19 (*p* = 0.31). Likewise, 10 (45.45%) of the recruited women were on metformin monotherapy, and 12 (54.54%) were on metformin plus sitagliptin. A total of seven (70.00%) of the women on metformin monotherapy developed mild-moderate COVID-19 compared to three (30.00%) who developed severe COVID-19 (*p* = 0.03). Furthermore, two (16.66%) of the women on metformin plus sitagliptin developed severe COVID-19 compared to 10 (83.33%) who developed mild-moderate COVID-19 (*p* = 0.01). There was no sex difference for the onset of severe COVID-19 in T2DM patients on metformin monotherapy (*p* = 0.35). However, male T2DM patients on metformin plus sitagliptin tended to develop severe COVID-19 more than women (*p* = 0.03). Furthermore, women on metformin plus sitagliptin tended to develop mild-moderate COVID-19 compared to men (*p* = 0.02), as shown in Table 4.

### 2.4. Assessment of COVID-19 Patients at the Time of Discharge

The clinical presentation of T2DM patients with severe COVID-19 was assessed at the time of discharge relative to outpatients of T2DM patients with mild-moderate COVID-19, as displayed in Table S3.

#### 2.4.1. Effects of Metformin versus Metformin plus Sitagliptin on the Anthropometric and Biochemical Variables at the Discharge Time

After 2–3 weeks from the time of hospitalization of T2DM patients with COVID-19, the assessment of patients was done before discharge. One patient with mild-moderate COVID-19 died of an unknown cause; three patients with severe COVID-19 died due to ARDS development. Therefore, 70 T2DM patients with mild-moderate COVID-19 and 26 T2DM patients with severe COVID-19 were analyzed. In mild-moderate COVID-19 patients, BMI, SBP, DBP, PP, and MAP were ameliorated in patients on metformin and sitagliptin relative to metformin monotherapy, but not significantly (*p* > 0.05). Regarding glycemic indices, FBG and FSI were lower, and IS was better in patients on metformin plus sitagliptin than metformin monotherapy (*p* < 0.01). HOMA2-IR, HbA1c, and β-cell function did not substantially differ in both groups (*p* > 0.05). Concerning the lipid profile and atherogenic indices, only TC, TG, HDL-C, and AI were ameliorated in COVID-19 patients on metformin plus sitagliptin compared to metformin monotherapy (*p* < 0.05). Furthermore, SaO2% did not significantly differ regarding current diabetic treatments (*p* > 0.05). Furthermore, WBC was reduced considerably in COVID-19 patients on metformin plus sitagliptin compared to metformin monotherapy (*p* = 0.001). However, lymphocyte and neutrophil % and NLR did not significantly differ in both groups (*p* > 0.05). Particularly, from the inflammatory biomarkers, only ferritin and D-dimer were reduced in COVID-19 patients on metformin plus sitagliptin as compared to metformin monotherapy (*p* < 0.05). Nevertheless, serum levels of CRP, LDH, and PCT did not differ regarding current diabetic treatments (*p* > 0.05) in COVID-19 patients with either mild-moderate or severe COVID-19 at time of discharge, as shown in Table 5.

**Figure 5:**
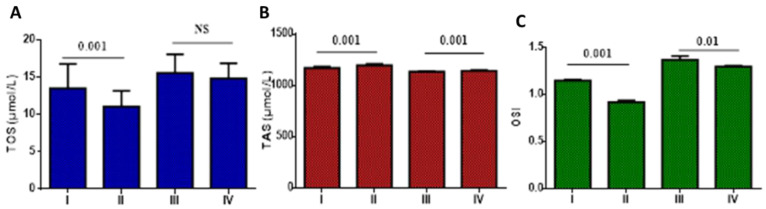
Effects of metformin monotherapy versus metformin plus sitagliptin treatment on the oxidative stress in T2DM patients with COVID-19: I: T2DM patients on metformin with mild-moderate COVID-19, II: T2DM patients on metformin plus sitagliptin with mild-moderate COVID-19, III: T2DM patients with severe COVID-19 on metformin monotherapy, IV: T2DM patients with severe COVID-19 on metformin plus sitagliptin. (**A**): TOS: total oxidant status; (**B**): TAS: total antioxidant status; (**C**): OSI: oxidative stress index.

Interestingly, oxidative stress was better ameliorated in COVID-19 patients on metformin plus sitagliptin relative to metformin monotherapy (*p* < 0.05). TOS was reduced from 13.52 ± 3.29 (µmol/L) in COVID-19 patients on metformin monotherapy to 11.09 ± 2.08 (µmol/L) in metformin plus sitagliptin as compared to metformin monotherapy (*p* = 0.001). However, TAS was increased from 1170.62 ± 11.09 (µmol/L) in COVID-19 patients on metformin monotherapy to 1196 ± 12.93 (µmol/L) in metformin plus sitagliptin relative to metformin monotherapy (*p* = 0.001). Therefore, OSI was reduced from 1.15 ± 0.01 in COVID-19 patients on metformin monotherapy to 0.92 ± 0.02 in patients on metformin plus sitagliptin (*p* = 0.001), as shown in Figure 5.

In severe COVID-19, BMI, SBP, DBP, and PP were ameliorated in patients on metformin plus sitagliptin treatment compared to metformin monotherapy, but not significantly (*p* > 0.05). MAP was reduced in patients on metformin plus sitagliptin treatment compared to metformin monotherapy (*p* = 0.002).

Concerning glycemic indices, FBG, β-cell function, FSI, and IS were better in patients on metformin plus sitagliptin relative to metformin monotherapy (*p* < 0.01). Though HOMA2-IR, and HbA1c did not substantially differ in both groups (*p* > 0.05). Regarding the lipid profile and atherogenic indices, TG, LDL, non-HDL-c, and AI were ameliorated in COVID-19 patients on metformin plus sitagliptin compared to metformin monotherapy (*p* < 0.05). Other indices were not significantly different in both treated groups (*p* > 0.05). Furthermore, SaO2% was not significantly different regarding current diabetic treatments (*p* > 0.05). In addition, CT scans and clinical scores were better in COVID-19 patients on metformin plus sitagliptin as compared to metformin monotherapy (*p* < 0.01).

Furthermore, WBC and lymphocyte % were significantly ameliorated in COVID-19 patients on metformin plus sitagliptin compared to metformin monotherapy (*p* < 0.05). However, neutrophil % and NLR did not significantly differ in both groups (*p* > 0.05). Remarkably, of the inflammatory biomarkers, only ferritin and D-dimer were reduced in COVID-19 patients on metformin plus sitagliptin relative to metformin monotherapy (*p* < 0.05). However, serum levels of CRP, LDH, and PCT did not differ regarding current diabetic treatments (*p* > 0.05), as shown in Table 5.

#### 2.4.2. Gender Assessment at the Time of Discharge

At the time of discharge, in 96 T2DM patients with COVID-19, the number of men was 74 (77.08%) compared to 22 (21.56%) recruited women (*p* = 0.001). A total of 51 (53.12%) of the men with COVID-19 were on metformin monotherapy compared to 33 (44.59%) on metformin plus sitagliptin (*p* = 0.33). A total of 33 (80.48%) of the men with COVID-19 who were on metformin monotherapy developed severe COVID-19 compared to 8 (19.51%) that developed mild-moderate COVID-19 (*p* = 0.01). In men with COVID-19 on metformin plus sitagliptin, 13 (39.39%) developed severe COVID-19 compared to 20 (60.60%) that developed mild-moderate COVID-19 (*p* = 0.32). Likewise, 10 (45.45%) of the recruited women were on metformin monotherapy, and 12 (54.54%) were on metformin plus sitagliptin. A total of seven (70.00%) of the women on metformin monotherapy developed mild-moderate COVID-19 compared to three (30.00%) that developed severe COVID-19 (*p* = 0.03). Furthermore, two (16.66%) of the women on metformin plus sitagliptin developed severe COVID-19 compared to 10 (83.33%) who developed mild-moderate COVID-19 (*p* = 0.01). There was no sex difference for developing severe COVID-19 in T2DM patients on metformin monotherapy (*p* = 0.29). However, male T2DM patients on metformin plus sitagliptin tended to develop severe COVID-19 more than women (*p* = 0.04). Furthermore, women on metformin plus sitagliptin tended to develop mild-moderate COVID-19 compared to men (*p* = 0.04), as shown in Table 6.

## 3. Discussion

The present study illustrated the potential effect of COVID-19 on the cardiometabolic and inflammatory biomarkers in T2DM patients. The possible therapeutic benefit of metformin and/or metformin plus sitagliptin on COVID-19 regarding inflammatory and oxidative stress complications were evaluated. Demographic characteristics of T2DM patients with COVID-19 showed that many of the recruited COVID-19 patients were of male sex at middle age. It has been shown that the male sex is a potential predisposing factor for developing severe COVID-19 disease because of the high level of testosterone hormone [17]. Moreover, COVID-19 can affect the pathogenesis and complications of T2DM. Unfortunately, T2DM COVID-19 patients are connected with high severity and mortality [5]. Higher expression of ACE2 in T2DM may increase the entry of SARS-CoV-2 [18]. In our study, most T2DM COVID-19 patients had high-level inflammatory biomarkers, including CRP, LDH, ferritin, and PCT [19].

Moreover, COVID-19 can provoke the development of coagulopathy and could be attributed to the direct cytopathic role of SARS-CoV-2 infection, which could be linked to the high pro-inflammatory cytokines [20]. Furthermore, oxidative stress is high in T2DM patients due to hyperglycemia-induced reactive oxygen species (ROS) generation and linked inflammatory disorders [21]. In COVID-19, oxidative stress is augmented due to the overproduction of ROS and decreased endogenous antioxidant capacity [22]. Exaggerated oxidative stress in T2DM COVID-19 patients increases the risk of inflammatory and coagulation disorders [22]. These agreed with the present study’s findings that revealed rising TOS and OSI with a decline of TAS in T2DM COVID-19 patients.

Moreover, severe COVID-19 may induce the development of IR and of new-onset DM [17]. It has been observed that COVID-19 is related to hyperglycemia [23]. Concisely, SARS-CoV-2 binds to ACE2, which is expressed in a very high percentage in the pancreatic cells, thus API will occur with insulin secretion impairment and hyperglycemia development, worsening the condition if DM is preexistent [24].

Furthermore, increased levels of TNF-α and IL-6 could result in peripheral IR, pancreatic β-cell function impairment, and a decrease in insulin secretion [25,26]. The prolonged hyperglycemia may cause the COVID-19 condition to deteriorate via increasing the entry of SARS-CoV-2 to the pancreatic β-cell by binding to ACE2 receptors [27]. 

In the present study, IR was increased while insulin sensitivity and pancreatic β-cell function were distorted in T2DM patients with COVID-19 at the time of admission. Treatment with metformin or metformin plus sitagliptin ameliorates peripheral IR, insulin sensitivity, and pancreatic β-cell function in COVID-19 patients. However, this effect did not significantly differ in mild-moderate COVID-19 patients on metformin compared to metformin plus sitagliptin. Nevertheless, the pancreatic β-cell function was ameliorated in severe COVID-19 patients on metformin plus sitagliptin compared to metformin monotherapy [28]. Such an issue might be attributed to the synergism among metformin and sitagliptin on pancreatic β-cell function [29]. Of note, IR and endothelial dysfunction are alleviated by the action of peroxisome and proliferative activated receptor gamma (PPARγ) in T2DM [28]. 

PPARγ agonist therapy in T2DM patients can preserve a healthy vasculature whilst decreasing the complications [28]. It has been hypothesized that pioglitazone could decrease the inflammation in COVID-19 patients at least in those who complain of T2DM [29]. Therefore, modulation of peripheral IR by PPARγ agonists in virtue of their anti-inflammatory activity may reduce COVID-19 severity in T2DM patients. Notoriously, different studies confirmed that metformin activates PPARγ with a subsequent improvement of IR [30,31,32]. 

Initial assessment showed that 55 (54.45%) T2DM COVID-19 patients were on metformin monotherapy. About 41 (74.54%) of them presented with mild-moderate COVID-19 compared to 14 (25.45%) of them who developed severe COVID-19 (*p* = 0.001). This finding suggests a protective role of metformin against the development of COVID-19 severity. It has been shown that metformin is effective in reducing disease severity [33]. Metformin has been observed to inhibit the binding of SARS-CoV-2 to the ACE2 by inducing phosphorylation of ACE2. Metformin also regulates the immune response through the induction development of anti-inflammatory regulatory T cells (Treg) and alternative macrophages [34]. In addition, metformin inhibits the expression of inflammatory signaling pathways, including NLRP3 inflammasome and NF-κB activated in COVID-19 [35]. 

Of note, metformin, an antidiabetic agent, may have pleiotropic anti-inflammatory and antioxidant effects [36], which might reduce SARS-CoV-2-triggered hyperinflammation and oxidative stress. Thus, metformin’s anti-inflammatory and antioxidant impacts can explain the reduction of inflammatory and oxidative biomarkers in the present study with further amelioration of radiological and clinical scores. Moreover, the experimental studies demonstrated that the administration of metformin could reduce the risk of ALI/ARDS induced by lipopolysaccharides (LPS) and paraquat [37]. These observations may explain the improvement effect of metformin on oxygenation percentage and radiological resolution in T2DM patients with severe COVID-19. Furthermore, metformin reduced D-dimer serum levels significantly in T2DM patients with severe COVID-19 at the time of discharge. A recent analysis of hospitalized T2DM COVID-19 patients revealed that metformin users were associated with lower thrombotic complications, as evidenced by low D-dimer serum levels [38]. Xin and colleagues confirmed that metformin could attenuate thrombosis risk by impeding platelet activation and the release of mitochondrial DNA in rats [39]. These verdicts may explain the potential lowering metformin effect on the D-dimer levels in T2DM COVID-19 patients.

Herein, the pressure profile of T2DM COVID-19 patients was reduced mainly in severe patients compared to the controlled T2DM patients. The reduction of blood pressure in severe COVID-19 patients may be due to dehydration caused by vomiting and diarrhea. Septic shock and dysautonomia may also develop in severely affected COVID-19 patients [40,41]. 

Remarkably, treatment with metformin plus sitagliptin significantly improved most glycaemic indices in T2DM COVID-19 patients. Moreover, T2DM COVID-19 patients on metformin plus sitagliptin therapy illustrated non-significant differences in cardiac index, TC, HDL, non-HDL, and NLR in severe T2DM COVID-19 patients as compared with mild-moderate T2DM COVID-19 patients. These annotations may explain the protective effects of this combination against the propagation of COVID-19 severity. Furthermore, oxidative stress was more evident in T2DM patients with severe COVID-19 at admission time and assessment. At the end of the treatment of the T2DM patients with COVID-19 on metformin plus sitagliptin therapy, one patient died with a 2.17% mortality rate, which was not significantly different compared to that of metformin monotherapy (*p* = 0.25).

Most anthropometric and pressure profiles and inflammatory and oxidative stress biomarkers were significantly lessened compared to time assessment and hospitalization. Of interest, clinical and CT scan scores were reduced in T2DM patients with severe COVID-19. The results of the present study confirmed metformin plus sitagliptin with standard therapy was influential in reducing COVID-19 severity. 

It has been proposed that DPP4 could be a possible entry point for SARS-CoV-2, though this finding is controversial, and there is not enough data to support this notion. However, blocking this receptor with sitagliptin may reduce the pathogenesis and infectivity of SARS-CoV-2 infection [42]. It has been hypothesized that inhibition of DPP4 by sitagliptin may reduce the propagation of hyperinflammation and cytokine storm in COVID-19 [43,44]. Furthermore, sitagliptin, through the inhibition of DPP4, increases circulating levels of glucagon-like peptide-1 (GLP-1), which has been shown to reduce inflammatory changes in COVID-19. These findings may explain the potent anti-inflammatory effects of sitagliptin. 

In this study, T2DM patients with mild-moderate COVID-19 on metformin plus sitagliptin illustrated more favorable effects on some biomarkers than those treated by metformin monotherapy. In T2DM patients with severe COVID-19 on metformin plus sitagliptin, there were more significant effects on some biomarkers of COVID-19 severity as compared with metformin monotherapy. Interestingly, clinical, and radiological scores were highly improved in T2DM patients with severe COVID-19 on metformin plus sitagliptin compared to those treated by metformin monotherapy.

These findings suggest that metformin plus sitagliptin therapy was more effective than metformin monotherapy in treating mild-moderate and severe COVID-19 in T2DM patients. To our knowledge, there is no reported study for this combination in treating COVID-19 T2DM patients, mainly in the severe form. Most of the severely affected COVID-19 T2DM patients are managed by insulin therapy for more strict blood glucose control [45]. The combination of metformin and sitagliptin may lead to additive or synergistic anti-inflammatory and antioxidant impacts [46]. These findings can explain the potent anti-inflammatory and antioxidant impacts compared to metformin monotherapy in COVID-19 T2DM patients. Notably, both metformin and sitagliptin have protective effects against ALI/ARDS development [47]. Thus, in this study, this combination produced more beneficial effects through the mitigation of clinical and CT scan scores as compared with metformin in COVID-19 T2DM patients. These findings suggest that metformin and sitagliptin has pulmoprotective properties against the development of AL/ARDS in COVID-19, as evidenced by the amelioration of lung CT scan scores and clinical outcomes.

Sitagliptin may increase growth and differentiation factor 15 (GDF15) through the augmentation of the GLP-1-dependent pathway [37]. Furthermore, metformin also improves the expression of GDF15 [48]. Therefore, the GLP-1-dependent pathway might be the possible mechanism linking the effect of metformin and sitagliptin in mitigating COVID-19 severity. However, GLP-1 and GDF15 serum levels were not evaluated in the present study to confirm the synergistic effects of metformin and sitagliptin on GLP-1 and GDF15 levels.

Of note, an extensive search of the literature was performed to analyze the potential pharmacokinetic and pharmacodynamic interactions between metformin and sitagliptin. Metformin and sitagliptin may be administered together, either separately or in a fixed-dose combination [45]. Metformin and sitagliptin are not subjected to pharmacokinetic drug–drug interactions. Their co-administration, either individually or in a fixed-dose combination, improves blood glucose control more potently than either compound separately, without hypoglycemia and increasing metformin-related gastrointestinal side effects [45]. Furthermore, it has been shown that metformin could enhance the biological effect of GLP-1 by increasing GLP-1 secretion, suppressing DPP-4 activity, and upregulating the expression of the GLP-1 receptor in pancreatic β-cells [46]. These findings suggest the safety profile of this combination with synergistic beneficial effects on glucose homeostasis. Randomized, single-dose, crossover studies conducted in healthy adults showed a lack of clinically meaningful pharmacokinetic interactions between metformin and sitagliptin as well as with other drugs, such as glimepiride and simvastatin [47].

Notoriously, this study showed that most T2DM women on metformin plus sitagliptin develop mild-moderate COVID-19, suggesting gender differences in response to this combination. A case-control study demonstrated that DPP-4 gene polymorphism was more evident in women compared to men [49]. Higher polymorphism of the DPP-4 gene in women may explain a higher response of T2DM women to the effect of DPP4 inhibitors [49]. This may explain why T2DM women on sitagliptin therapy develop mild-moderate COVID-19 compared to metformin monotherapy. The current study confirmed the gender-specific effect of metformin alone or with sitagliptin for the first time against the development of COVID-19 in T2DM patients.

Taken together, the current study confirmed, for the first time, that the metformin plus sitagliptin combination add-on standard therapy was more effective than metformin monotherapy in the treatment of T2DM patients with COVID-19. Further preclinical, clinical, and large-scale studies are required to verify this combination’s protective effects in both diabetic and diabetic COVID-19 patients. 

## 4. Materials and Methods

### 4.1. Materials and Chemicals

A glucophage tab (metformin 850 mg, Pioneer, Sulaymaniyah, Iraq) and Janumet (metformin 1000 mg + sitagliptin 50 mg, Merck, Rahway, NJ, USA) were used in this study. ELISA kits were obtained from MyBioSource, San Diego, CA, USA.

### 4.2. Patients

A total number of 112 T2DM patients with COVID-19 ranging in age from 44 to 62 years were recruited from Al-Shiffa Medical Center from the outpatient clinic and in-hospital wards compared with 78 T2DM patients without COVID-19 aged 42–56 years as controls. The selection of cases with COVID-19 and T2DM was done according to the international quid for the diagnostic criteria of COVID-19 and the American Diabetic Association, respectively [50,51]. 

### 4.3. Experimental Protocol

The present case-control, single-center cohort study was conducted in the Department of Clinical Pharmacology and Therapeutics, College of Medicine, Al-Mustansiriyiah University, in cooperation with Al-Shiffa Medical Center Bagdad, Iraq, from March to July 2021 in collaboration with Egypt (Ethics Committee research number R249, Fayoum University). This study was permitted by the Scientific Jury and Ethical Committee Editorial Board in the College of Medicine, Al-Mustansiriyiah University. Ethical approval No. 327JRT on 22 January 2021. 

Both patients and matched control were allocated into four groups:

- Group A: T2DM patients with COVID-19 (10 women + 50 men) on metformin treatments 850 mg twice daily plus standard therapy (n = 60).
- Group B: T2DM patients with COVID-19 (12 women + 40 men) on metformin (1000 mg/daily) plus sitagliptin (50 mg/ daily) plus standard therapy (n = 52).
- Group C: T2DM patients without COVID-19 (15 women + 25 men) on metformin treatments 850 mg twice (n = 40).
- Group D: T2DM patients without COVID-19 (13 women + 25 men) on metformin (1000 mg/ daily) plus sitagliptin (50 mg/ daily) (n = 38).

The investigation duration was 2–3 weeks. The standard therapy utilized in COVID-19 management was supportive treatments, such as analgesic, antipyretic, ivermectin (Merck, Rahway, NJ, USA) 6 mg/day, famotidine (Merck, Rahway, NJ, USA) 40 mg/day, and azithromycin (Merck, Rahway, NJ, USA) 500 mg/day. However, the standard therapy for hospitalized cases with severe COVID-19 was supportive treatments, such as analgesic, antipyretic, as well as prophylactic antibiotics, ivermectin 6 mg/day, famotidine 40 mg/day, azithromycin 500 mg/day, favipiravir (Merck, Rahway, NJ, USA) 1800 mg/day, subcutaneous enoxaparin (Merck, Rahway, NJ, USA) 4000–6000 iu/day, intravenous remdesivir (Merck, Rahway, NJ, USA) 100 mg/day, and oxygen supplementations.

### 4.4. Inclusion Criteria

T2DM patients with or without COVID-19 on metformin monotherapy or metformin plus sitagliptin were involved in this study.

### 4.5. Exclusion Criteria

T2DM cases with other diabetic pharmacotherapies, chronic kidney disorders, severe hepatic insufficiency, autoimmune and connective tissue diseases, mental and psychiatric disorders, pregnancy, and lactation were excluded from this study.

### 4.6. Anthropometric Measurements

A specific equation calculated body mass index (BMI) [8],

BMI = weight (kg)/height (m^2^)

Both patients’ and controls’ systolic blood pressure (SBP) and diastolic blood pressure (DBP) were monitored [8].

### 4.7. Serological and Biochemical Investigations

After overnight fasting, ten milliliters of blood were taken and centrifuged. Real-time PCR (RT-PCR) (COVID-19, Altona Diagnostics, Hamburg, Germany) was performed for any T2DM patient with suspected symptoms of COVID-19. Furthermore, a COVID-19 test (Altona Diagnostics, Hamburg, Germany) was done for the detection of anti-SARS-CoV-2 antibodies IgM (indicating a recent infection) and IgG (indicating a previous condition). 

A complete blood count (CBC) for each suspected COVID-19 patient was performed by a CBC auto-analyzer (Xinke-Zhejiang Medical Technology, Jinhua, China). Fasting blood glucose (FBG) was assessed using an automated colorimetric assay (Glucose test, BioSystem, Lexington, MA, USA). Glycated hemoglobin (HbA1c) was measured by HbA1c Rapid Quantitative Test (Cipalstraat, Geel, Belgium). Fasting serum insulin (FSI) was assessed by the ELISA kit method. In addition, the homeostatic model for the evaluation of IR (HOMA2-IR) was applied for the estimation state of IR, β-cell function, and insulin sensitivity [7].

Further, lipid profiles, including total cholesterol (TC), triglyceride (TG), and high-density lipoprotein (HDL), were estimated by specific ELISA kit methods (Cambridge, UK). The low-density lipoprotein (LDL) was measured according to the Friedewald formula [7,52,53].

Likewise, the levels of the inflammatory biomarkers, such as C-reactive protein (CRP), ferritin, procalcitonin, lactate dehydrogenase (LDH), a lung injury biomarker, and D-dimer of coagulopathy were determined using ELISA kits following the instructions of the manufacturer. Furthermore, the oxidative stress index (OSI) was calculated using an ELISA kit [8].

### 4.8. Assessment of Pulmonary Radiological Findings

Posterior-anterior chest X-ray and lung CT scan were conducted on COVID-19 patients to detect lung injury, mainly the bilateral ground-glass opacity (GGO). CT scan results were recorded regarding the lung involvement percentage [54]. The scores were: (I) lung involvement < 5%, (II) lung involvement 5–25%, (III) lung involvement 26–50%, (IV) lung involvement 51–75%, and (V) lung involvement > 75%.

### 4.9. Assessment of Clinical Outcomes

As presented in Table 7, clinical scoring of hospitalized COVID-19 patients were assessed at the discharge time [52]. This clinical scoring system is ranged from I to VII scores.

### 4.10. Data Analysis

The current study’s data analysis was analyzed using Graph pad prism 8 (San Diego, CA, USA). Results of the present study were revealed as mean ± standard deviation (SD). An ANOVA test was applied to detect the statistical difference among more than three groups. The level of significance was regarded when the *p*-value < 0.05.

## 5. Conclusions

SARS-CoV-2 is the cause of the development of COVID-19, which causes pulmonary and extra-pulmonary complications. SARS-CoV-2 attaches to ACE2 receptors. Furthermore, SARS-CoV-2 can bind DPP4 to a lesser extent than ACE2, causing hyperinflammation and oxidative stress. T2DM is a risk factor for SARS-CoV-2 infection and is linked with increasing the severity of COVID-19. Metformin is an antidiabetic drug belonging to the biguanide class with anti-hyperglycaemic activity. Metformin is commonly used to manage T2DM, mainly in overweight and obese patients. Sitagliptin is an antidiabetic drug from the DPP4 inhibitors family that acts by increasing insulin secretion with an inhibition of glucagon secretion.

A combination of sitagliptin and metformin was permitted for the management of T2DM. In the present case-control cohort study, T2DM patients with COVID-19 had severe changes in the inflammatory biomarkers and cardiometabolic profile compared to controls, suggesting that SARS-CoV-2 infection induces IR and pancreatic β cell dysfunction. T2DM patients on metformin monotherapy or metformin plus sitagliptin develop less COVID-19 severity. However, metformin plus sitagliptin was more effective than metformin monotherapy in T2DM patients with COVID-19, as evidenced by the mitigation of oxidative stress, CT scan score, and clinical outcomes. Thus, we can conclude the protective effects of this combination against the development of COVID-19 severity, as most T2DM COVID-19 patients develop mild-moderate forms. The combination of metformin plus sitagliptin may lead to more beneficial effects than metformin monotherapy.

Nevertheless, serum levels of CRP, LDH, and PCT have not differed regarding current diabetic treatments in both metformin monotherapy and metformin plus sitagliptin. Remarkably, male T2DM patients on metformin plus sitagliptin tend to develop severe COVID-19 more than female patients. Taken together, the current study confirmed, for the first time, that the metformin plus sitagliptin combination add-on standard therapy was more effective than metformin monotherapy in T2DM patients with COVID-19. Further studies are required to verify the protective effects of this combination both in diabetic and diabetic COVID-19 patients.

## Figures and Tables

**Table 1:** Metformin effects on the anthropometric and biochemical variables in T2DM with COVID-19 relative to T2DM patients.

Variables	Group I	Group II	Group III	A	B	C	ANOVA
	(*n* = 40)	(*n* = 41)	(*n* = 14)				
SBP (mmHg)	143.67 ±11.56	140.41 ± 10.93	140.78 ± 9.53	NS	NS	NS	0.38
DBP (mmHg)	89.85 ±7.33	82.56 ± 5.85	73.91 ± 6.61	0.0001	0.0001	0.0001	0.0001
MAP (mmHg)	107.80 ± 4.12	101.80 ± 3.99	96.2 ± 4.41	0.01	0.0001	0.0001	0.0001
PP (mmHg)	53.81 ± 3.07	57.84 ± 4.67	66.87 ± 3.94	0.01	0.0001	0.0001	0.0001
FBG (mg/dL)	144.72 ± 6.92	159.62 ± 8.46	187.91 ± 9.54	0.01	0.0001	0.0001	0.0001
HbA1c (%)	7.23 ± 1.31	7.07 ± 1.55	7.51 ± 1.31	NS	NS	NS	0.59
FSI (µIU/mL)	19.85 ± 6.97	19.47 ± 8.38	27.08 ± 8.44	NS	0.01	0.006	0.006
HOMA2-IR	2.81 ± 1.04	2.82 ± 1.07	3.98 ± 1.06	NS	0.001	0.001	0.006
β-cell function (%)	75.00 ± 6.84	61.9 ± 5.90	56.9 ± 5.33	0.0001	0.0001	0.03	0.0001
IS (%)	35.60 ± 6.98	35.62 ± 4.07	25.13 ± 3.02	NS	0.0001	0.0001	0.0001
TC (mg/dL)	198.68 ± 12.81	141.64 ± 9.51	130.91 ± 5.82	0.0001	0.0001	0.0001	0.0001
TG (mg/dL)	223.95 ± 14.78	230.61 ± 13.20	274.12 ± 6.11	NS	0.0001	0.0001	0.0001
HDL-c (mg/dL)	43.61 ± 7.33	37.05 ± 6.04	31.63 ± 6.81	0.0001	0.0001	0.02	0.0001
LDL (mg/dL)	110.3 ± 6.39	58.5 ± 4.38	44.50 ± 4.05	0.0001	0.0001	0.01	0.0001
VLDL (mg/dL)	44.79 ± 4.33	46.12 ± 4.11	54.82 ± 3.61	NS	0.0001	0.0001	0.0001
Non-HDL-c (mg/dL)	155.07 ± 9.57	104.59 ± 9.39	99.28 ± 6.03	NS	0.0001	NS	0.0001
AI	0.71 ± 0.01	0.77 ± 0.02	0.93 ± 0.04	0.0001	NS	0.03	0.0001
AC	5.55 ± 1.02	2.82 ± 1.04	3.13 ± 1.02	0.0001	0.0001	NS	0.0001
CRR	4.55 ± 1.72	3.82 ± 1.02	4.13 ± 1.06	NS	NS	NS	0.06
CVRI	5.36 ± 2.29	6.22 ± 2.99	8.66 ± 3.06	NS	0.005	0.01	0.0009
SaO2 (%)	99.86 ± 1.23	98.89 ± 1.76	90.04 ± 3.15	NS	0.0001	0.0001	0.0001
CT scan score (%)	…………..	3.82 ± 1.27	40.61 ± 3.85	…….	……..	0.0001	……..
WBC (10^3^/µL)	8.09 ± 2.41	10.90 ± 3.04	16.38 ± 4.57	0.03	0.0001	0.0001	0.0001
Neutrophils (%)	75.91 ± 6.80	79.31 ± 8.59	88.53 ± 7.18	NS	0.0001	0.0001	0.0001
Lymphocytes (%)	24.73 ± 3.05	20.82 ± 4.65	13.62 ± 5.71	0.002	0.0001	0.0001	0.0001
NLR	3.06 ± 1.09	3.80 ± 1.99	6.51 ± 2.04	NS	0.0001	0.0001	0.0001
CRP (mg/L)	7.02 ± 2.06	10.42 ± 3.11	21.72 ± 5.19	0.02	0.0001	0.0001	0.0001
Ferritin (ng/mL)	190.38 ± 13.97	257.31 ± 15.33	494.31 ± 11.39	0.0001	0.0001	0.0001	0.0001
LDH (U/L)	110.83 ± 9.92	267.68 ± 11.97	382.68 ± 12.11	0.0001	0.0001	0.04	0.0001
D-dimer (ng/mL)	200.16 ± 10.11	283.68 ± 13.01	463.82 ± 10.11	0.0001	0.0001	0.02	0.0001
PCT (ng/mL)	0.06 ± 0.01	0.18 ± 0.03	0.26 ± 0.05	0.0001	0.0001	0.003	0.0001

I: T2DM patients on metformin, II: T2DM with mild-moderate COVID-19 on metformin, III: T2DM with severe COVID-19 on metformin patients, A: I vs. II, B: I vs. III, C: II vs. III. NS: not significant, BMI: body mass index, SBP: systolic blood pressure, DBP: diastolic blood pressure, MAP: mean arterial pressure, PP: pulse pressure, FBG: fasting blood glucose, HbA1c: glycated hemoglobin, FSI: fasting serum insulin, HOMA2-IR: a homeostatic model for assessment of IR, TC: total cholesterol, TG: triglyceride, HDL: high-density lipoprotein, LDL: low-density lipoprotein, VLDL: very-low-density lipoprotein AI: atherogenic index, AC: atherogenic coefficient, CRR: cardiac risk ratio, CVRI: cardiovascular risk index, CRP: C-reactive protein, PCT: procalcitonin, LDH: lactate dehydrogenase.

**Table 2:** Impacts of metformin plus sitagliptin on the anthropometric and biochemical variables in T2DM patients with COVID-19 compared to T2DM patients.

Variables	Group I	Group II	Group III	A	B	C	ANOVA
	(*n* = 38)	(*n* = 30)	(*n* = 16)				
BMI (kg/m^2^)	30.66 ± 4.63	31.74 ± 3.99	32.82 ± 3.99	NS	NS	NS	0.22
SBP (mmHg)	145.85 ± 11.63	139.69 ± 11.81	137.06 ± 10.59	NS	0.03	NS	0.01
DBP (mmHg)	90.01 ± 6.89	81.75 ± 6.32	68.61 ± 8.64	0.0001	0.0001	0.0001	0.0001
MAP (mmHg)	108.60 ± 4.96	101.10 ± 4.03	91.40 ± 4.32	0.0001	0.0001	0.0001	0.0001
PP (mmHg)	55.83 ± 3.45	57.94 ± 4.69	68.45 ± 5.19	NS	0.0001	0.0001	0.0001
FBG (mg/dL)	133.06 ± 7.22	148.53 ± 6.85	157.91 ± 7.54	0.0001	0.0001	0.0002	0.0001
HbA1c (%)	7.01 ± 1.99	7.52 ± 1.21	7.02 ± 1.44	NS	NS	NS	0.4
FSI (µIU/mL)	17.04 ± 8.11	18.11 ± 6.94	22.03 ± 8.17	NS	NS	NS	0.09
HOMA2-IR	2.38 ± 1.03	2.58 ± 1.05	3.15 ± 1.08	NS	0.04	NS	0.05
β-cell function (%)	77.7 ± 8.93	66.7 ± 6.47	69.50 ± 7.05	0.0001	0.0001	NS	0.0001
IS (%)	42.0 ± 5.06	38.7 ± 6.33	31.70 ± 4.39	0.03	0.0001	0.0002	0.0001
TC (mg/dL)	183.07 ± 11.91	139.06 ± 9.61	133.21 ± 6.04	0.0001	0.0001	NS	0.0001
TG (mg/dL)	214.51 ± 12.79	231.93 ± 13.74	264.33 ± 9.33	0.0001	0.0001	0.0001	0.0001
HDL-c (mg/dL)	44.94 ± 7.08	38.05 ± 6.44	33.93 ± 6.51	0.0002	0.0001	NS	0.0001
LDL (mg/dL)	95.2 ± 4.99	54.2 ± 4.22	46.40 ± 3.22	0.0001	0.0001	0.03	0.0001
VLDL (mg/dL)	42.90 ± 4.99	46.38 ± 4.69	52.86 ± 5.31	0.01	0.0001	0.0001	0.0001
Non-HDL-c (mg/dL)	138.13 ± 8.19	101.01 ± 7.73	99.28 ± 5.81	0.0001	0.0001	NS	0.0001
AI	0.67 ± 0.02	0.78 ± 0.01	0.89 ± 0.02	NS	NS	0.0001	0.0001
AC	3.07 ± 2.66	2.65 ± 1.22	2.92 ± 1.04	NS	NS	NS	0.68
CRR	4.07 ± 1.09	3.65 ± 1.33	3.92 ± 1.19	NS	NS	NS	0.36
CVRI	4.77 ± 2.56	6.09 ± 2.58	7.79 ± 3.09	NS	0.008	NS	0.001
SaO2 (%)	99.31 ± 2.21	98.05 ± 2.29	94.04 ± 2.02	NS	0.0001	0.0001	0.0001
CT scan (%)	…………..	3.01 ± 1.12	35.17 ± 2.36	…….	……..	0.0001	……….
WBC (10^3^/µL)	8.79 ± 2.81	10.57 ± 3.55	14.29 ± 3.24	NS	0.0001	0.0008	0.0001
Neutrophils (%)	73.31 ± 5.41	77.31 ± 8.04	78.91 ± 6.08	0.03	0.01	NS	0.006
Lymphocytes (%)	27.06 ± 3.63	19.03 ± 4.85	17.51 ± 4.61	0.0001	0.0001	NS	0.0001
NLR	2.70 ± 1.22	4.06 ± 1.84	4.50 ± 1.97	0.002	0.001	NS	0.0001
CRP (mg/L)	6.45 ± 2.09	11.32 ± 3.04	20.41 ± 6.04	0.01	0.0001	0.0001	0.0001
Ferritin (ng/mL)	187.27 ± 14.97	249.36 ± 14.11	464.62 ± 12.78	0.0001	0.0001	0.0001	0.0001
LDH (U/L)	113.31 ± 10.03	272.41 ± 11.02	372.38 ± 13.81	0.0001	0.0001	0.0001	0.0001
D-dimer (ng/mL)	207.85 ± 11.61	294.83 ± 12.54	413.05 ± 14.73	0.0001	0.0001	0.0001	0.0001
PCT (ng/mL)	0.09 ± 0.02	0.16 ± 0.03	0.21 ± 0.02	0.0001	0.0001	0.0001	0.0001

I: T2DM patients on metformin plus sitagliptin, II: T2DM patients with mild-moderate COVID-19 on metformin plus sitagliptin, III: T2DM patients with severe COVID-19 on metformin plus sitagliptin, A: I vs. II, B: I vs. III, C: II vs. III. NS: not significant, BMI: body mass index, SBP: systolic blood pressure, DBP: diastolic blood pressure, MAP: mean arterial pressure, PP: pulse pressure, FBG: fasting blood glucose, HbA1c: glycated hemoglobin, FSI: fasting serum insulin, HOMA2-IR: a homeostatic model for assessment of IR, TC: total cholesterol, TG: triglyceride, HDL: high-density lipoprotein, LDL: low-density lipoprotein, VLDL: very-low-density lipoprotein AI: atherogenic index, AC: atherogenic coefficient, CRR: cardiac risk ratio, CVRI: cardiovascular risk index, CRP: C-reactive protein, PCT: procalcitonin, LDH: lactate dehydrogenase, TOS: total oxidant status, TAS: total antioxidant status, OSI: oxidative stress index.

**Table 3:** Impacts of metformin and metformin plus sitagliptin on the anthropometric and biochemical variables in T2DM patients with COVID-19.

Variables	Mild-Moderate COVID-19		*p*	Severe COVID-19		*p*
	Group I (*n* = 30)	Group II (*n* = 41)		Group III (*n* = 14)	Group IV (*n* = 16)	
BMI (kg/m^2^)	31.83 ± 4.22	31.74 ± 3.99	NS	35.09 ± 4.99	32.82 ± 3.99	NS
SBP (mmHg)	140.41 ± 10.93	139.69 ± 11.81	NS	140.78 ± 9.53	137.06 ± 10.59	NS
DBP (mmHg)	82.56 ± 5.85	81.75 ± 6.32	NS	73.91 ± 6.61	68.61 ± 8.64	NS
MAP (mmHg)	101.80 ± 3.99	101.10 ± 4.03	NS	96.20 ± 4.41	91.40 ± 4.32	0.005
PP (mmHg)	57.84 ± 4.67	57.94 ± 4.69	NS	66.87 ± 3.94	68.45 ± 5.19	NS
FBG (mg/dL)	159.62 ± 8.46	148.53 ± 6.85	0.001	187.91 ± 9.54	157.91 ± 7.54	0.001
HbA1c (%)	7.07 ± 1.55	7.52 ± 1.21	NS	7.51 ± 1.31	7.02 ± 1.44	NS
FSI (µIU/mL)	19.47 ± 8.38	18.11 ± 6.94	NS	27.08 ± 8.44	22.03 ± 8.17	NS
HOMA2-IR	2.82 ± 1.07	2.58 ± 1.05	NS	3.98 ± 1.06	3.15 ± 1.08	NS
β-cell function (%)	61.9 ± 5.90	66.7 ± 6.47	0.001	56.9 ± 5.33	69.50 ± 7.05	0.001
IS (%)	35.62 ± 4.07	38.7 ± 6.33	0.01	25.13 ± 3.02	31.70 ± 4.39	0.001
TC (mg/dL)	141.64 ± 9.51	139.06 ± 9.61	NS	130.91 ± 5.82	133.21 ± 6.04	NS
TG (mg/dL)	230.61 ± 13.20	231.93 ± 13.74	NS	274.12 ± 6.11	264.33 ± 9.33	0.002
HDL-c (mg/dL)	37.05 ± 6.04	38.05 ± 6.44	NS	31.63 ± 6.81	33.93 ± 6.51	NS
LDL (mg/dL)	58.5 ± 4.38	54.2 ± 4.22	0.001	44.50 ± 4.05	46.40 ± 3.22	NS
VLDL (mg/dL)	46.12 ± 4.11	46.38 ± 4.69	NS	54.82 ± 3.61	52.86 ± 5.31	NS
Non-HDL-c (mg/dL)	104.59 ± 9.39	101.01 ± 7.73	NS	99.28 ± 6.03	99.28 ± 5.81	NS
AI	0.77 ± 0.02	0.78 ± 0.01	NS	0.93 ± 0.04	0.89 ± 0.02	0.001
AC	2.82 ± 1.04	2.65 ± 1.22	NS	3.13 ± 1.02	2.92 ± 1.04	NS
CRR	3.82 ± 1.02	3.65 ± 1.33	NS	4.13 ± 1.06	3.92 ± 1.19	NS
CVRI	6.22 ± 2.99	6.09 ± 2.58	NS	8.66 ± 3.06	7.79 ± 3.09	NS
SaO2 (%)	98.89 ± 1.76	98.05 ± 2.29	NS	90.04 ± 3.15	94.04 ± 2.02	0.0002
CT scan score (%)	3.82 ± 1.27	3.01 ± 1.12	NS	40.61 ± 3.85	35.17 ± 2.36	0.001
WBC (10^3^/µL)	10.90 ± 3.04	10.57 ± 3.55	NS	16.38 ± 4.57	14.29 ± 3.24	NS
Neutrophils (%)	79.31 ± 8.59	77.31 ± 8.04	NS	88.53 ± 7.18	78.91 ± 6.08	0.0004
Lymphocytes (%)	20.82 ± 4.65	19.03 ± 4.85	NS	13.62 ± 5.71	17.51 ± 4.61	0.04
NLR	3.80 ± 1.99	4.06 ± 1.84	NS	6.51 ± 2.04	4.50 ± 1.97	0.01
CRP (mg/L)	10.42 ± 3.11	11.32 ± 3.04	NS	21.72 ± 5.19	20.41 ± 6.04	NS
Ferritin (ng/mL)	257.31 ± 15.33	249.36 ± 14.11	0.02	494.31 ± 11.39	464.62 ± 12.78	0.001
LDH (U/L)	267.68 ± 11.97	272.41 ± 11.02	NS	382.68 ± 12.11	372.38 ± 13.81	0.03
D-dimer (ng/mL)	283.68 ± 13.01	294.83 ± 12.54	0.0006	463.82 ± 10.11	413.05 ± 14.73	0.001
PCT (ng/mL)	0.18 ± 0.03	0.16 ± 0.03	NS	0.26 ± 0.05	0.21 ± 0.02	0.001

I: T2DM patients with mild-moderate COVID-19 on metformin, II: T2DM patients with mild-moderate COVID-19 on metformin plus sitagliptin, III: T2DM patients with severe COVID-19 on metformin, IV: T2DM patients with severe COVID-19 on metformin plus sitagliptin. NS: not significant, BMI: body mass index, SBP: systolic blood pressure, DBP: diastolic blood pressure, MAP: mean arterial pressure, PP: pulse pressure, FBG: fasting blood glucose, HbA1c: glycated hemoglobin, FSI: fasting serum insulin, HOMA2-IR: a homeostatic model for assessment of IR, TC: total cholesterol, TG: triglyceride, HDL: high-density lipoprotein, LDL: low-density lipoprotein, VLDL: very-low-density lipoprotein AI: atherogenic index, AC: atherogenic coefficient, CRR: cardiac risk ratio, CVRI: cardiovascular risk index, CRP: C-reactive protein, PCT: procalcitonin, LDH: lactate dehydrogenase, TOS: total oxidant status, TAS: total antioxidant status, OSI: oxidative stress index.

**Table 4:** Gender difference and COVID-19 severity regarding metformin or metformin plus sitagliptin.

Variables	Total	Men	Women	*p*
*n*	101 (100%)	79 (78.21%)	22 (21.78%)	0.001
On metformin only	55 (54.45%)	45 (56.96%)	10 (45.45%)	0.34
Mild-moderate	41 (74.54%)	34 (75.56%) *	7 (70.00%) **	0.59
Severe	14 (25.45%)	11 (24.44%)	3 (30.00%)	0.59
On metformin plus sitagliptin	46 (45.54%)	34 (43.03%)	12 (54.54%)	0.34
Mild-moderate	30 (65.21%)	20 (58.82%)	10 (83.33%) ***	0.02
Severe	16 (34.78)	14 (41.17%)	2 (16.66%)	0.03

* *p* < 0.05 as compared to severe COVID-19 men patients on metformin monotherapy, ** *p* < 0.05 as compared to severe COVID-19 women patients on metformin monotherapy, *** *p* < 0.05 as compared to severe COVID-19 women patients on metformin plus sitagliptin.

**Table 5:** Metformin effects versus metformin plus sitagliptin on anthropometric and biochemical variables in T2DM patients with COVID-19.

Variables	Mild-Moderate COVID-19		*p*	Severe COVID-19		*p*
	Group I (*n* = 40)	Group II (*n* = 30)		Group III (*n* = 11)	Group IV (*n* = 15)	
BMI (kg/m^2^)	31.97 ± 4.95	31.33 ± 3.21	NS	35.11 ± 4.32	32.74 ± 3.98	NS
SBP (mmHg)	141.83 ± 10.07	140.69 ± 10.11	NS	142.05 ± 9.38	139.89 ± 9.36	NS
DBP (mmHg)	80.78 ± 5.31	80.94 ± 5.39	NS	75.04 ± 6.93	76.03 ± 6.91 *	NS
MAP (mmHg)	101.13 ± 3.53	100.68 ± 4.72	NS	97.38 ± 4.91	91.40 ± 4.32 *	0.002
PP (mmHg)	61.05 ± 4.84 *	59.57 ± 4.22	NS	67.01 ± 3.41 *	68.49 ± 5.19	NS
FBG (mg/dL)	112.92 ± 7.03 *	103.99 ± 7.11 #	0.001	127.03 ± 6.91 *	117.56 ± 6.44 *	0.001
HbA1c (%)	7.07 ± 1.55	7.52 ± 1.21	NS	7.51 ± 1.31	7.02 ± 1.44	NS
FSI (µIU/mL)	12.06 ± 2.66 *	9.11 ± 2.61 #	0.01	17.08 ± 4.18 *	12.09 ± 5.36 *	0.01
HOMA2-IR	1.64 ± 0.25 *	1.22 ± 0.05 #	NS	2.36 ± 1.05 *	1.66 ± 1.08 *	NS
β-cell function (%)	84.3 ± 7.04 *	81. 8 ± 7.33 #	NS	84.8 ± 5.11 *	77.6 ± 5.09 *	0.001
IS (%)	61.00 ± 8.31 *	81.9 ± 8.91 #	0.01	42.3 ± 3.02 *	60.3 ± 4.05 #	0.001
TC (mg/dL)	160.05 ± 8.21 *	165.06 ± 8.99 #	0.01	167.21 ± 5.82 *	163.47 ± 5.94 #	NS
TG (mg/dL)	168.03 ± 9.10 *	161.73 ± 9.57 #	0.01	178.19 ± 4.22 *	174.64 ± 4.03 #	0.03
HDL-c (mg/dL)	43.05 ± 5.22 *	48.05 ± 5.92 #	0.03	40.44 ± 5.03 *	43.51 ± 5.81 #	NS
LDL (mg/dL)	83.4 ± 6.06 *	84.7 ± 4.73 #	NS	91.05 ± 4.68 *	85.00 ± 4.29 #	0.02
VLDL (mg/dL)	33.66 ± 5.21 *	32.34 ± 5.03 #	NS	35.63 ± 3.93 *	34.92 ± 5.31 #	NS
Non-HDL-c (mg/dL)	117.00 ± 8.05 *	117.01 ± 8.91 #	NS	126.77 ± 5.11 *	119.96 ± 5.06 #	0.03
AI	0.59 ± 0.01 *	0.52 ± 0.02 #	0.01	0.64 ± 0.03 *	0.60 ± 0.01 #	0.01
AC	2.71 ± 1.48 *	2.43 ± 1.29 #	NS	3.17 ± 1.09 *	2.79 ± 1.02 #	NS
CRR	3.71 ± 1.09 *	3.43 ± 1.33 #	NS	4.17 ± 1.02 *	3.79 ± 1.05 #	NS
CVRI	3.90 ± 1.92 *	3.35 ± 1.22 #	NS	4.45 ± 1.01 *	4.04 ± 1.22 #	NS
SaO2 (%)	99.91 ± 1.01	99.05 ± 1.04	NS	96.04 ± 3.21 *	97.09 ± 2.09 #	NS
CT scan score (%)	…………..	………….	…..	10.12 ± 2.01 *	4.01 ± 1.99 #	0.001
Clinical score (0–7)	……………	…………..	……	2.31 ± 1.05 *	1.03 ± 0.61 #	0.01
WBC (10^3^/µL)	8.39 ± 2.01 *	10.57 ± 3.55 #	0.001	11.38 ± 3.88 *	14.29 ± 3.24 #	0.04
Neutrophils (%)	75.91 ± 5.19 *	74.31 ± 5.09 #	NS	78.27 ± 3.19 *	77.91 ± 5.09 #	NS
Lymphocytes (%)	25.92 ± 3.65 *	26.03 ± 3.04 #	NS	20.69 ± 2.02	23.51 ± 3.65 #	0.02
NLR	2.92 ± 1.99 *	2.85 ± 1.84 #	NS	3.78 ± 2.55 *	3.31 ± 2.02 #	NS
CRP (mg/L)	3.21 ± 1.57 *	3.83 ± 1.51 #	NS	7.53 ± 2.19 *	8.25 ± 3.82 #	NS
Ferritin (ng/mL)	159.61 ± 7.82 *	149.36 ± 6.04 #	0.001	286.31 ± 6.04 *	260.62 ± 8.06 #	0.001
LDH (U/L)	257.68 ± 9.97 *	261.10 ± 10.33 #	NS	350.23 ± 11.12 *	359.22 ± 12.35 #	NS
D-dimer (ng/mL)	144.93 ± 8.55 *	131.02 ± 5.82 #	0.01	211.02 ± 6.88 *	201.05 ± 5.12 #	0.0002
PCT (ng/mL)	0.10 ± 0.01*	0.09 ± 0.01 #	NS	0.14 ± 0.03 *	0.11 ± 0.02 #	NS

I: T2DM patients with mild-moderate COVID-19 on metformin, II: T2DM patients with mild-moderate COVID-19 on metformin plus sitagliptin, III: T2DM patients with severe COVID-19 on metformin, IV: T2DM patients with severe COVID-19 on metformin plus sitagliptin. NS: not significant, BMI: body mass index, SBP: systolic blood pressure, DBP: diastolic blood pressure, MAP: mean arterial pressure, PP: pulse pressure, FBG: fasting blood glucose, HbA1c: glycated hemoglobin, FSI: fasting serum insulin, HOMA2-IR: a homeostatic model for assessment of IR, TC: total cholesterol, TG: triglyceride, HDL: high-density lipoprotein, LDL: low-density lipoprotein, VLDL: very-low-density lipoprotein AI: atherogenic index, AC: atherogenic coefficient, CRR: cardiac risk ratio, CVRI: cardiovascular risk index, CRP: C-reactive protein, PCT: procalcitonin, LDH: lactate dehydrogenase. * *p* < 0.05 as compared to the time of admission or assessment. # *p* < 0.05 as compared to the time of admission or assessment. The effects of metformin versus metformin plus sitagliptin on the oxidative stress at the discharge time is shown in Figure 5.

**Table 6:** Gender difference and COVID-19 severity regarding metformin or metformin plus sitagliptin.

Variables	Total	Men	Women	*p*
*n*	96 (100%)	74 (77.08%)	22 (21.91%)	0.001
On metformin only	51 (53.12%)	41 (55.40%)	10 (45.45%	0.41
Mild-moderate	40 (78.43%)	33 (80.48%) *	7 (70.00%) **	0.29
Severe	11 (21.56%)	8 (19.51%)	3 (30.00%)	0.29
On metformin plus sitagliptin	45 (46.87%)	33 (44.59%)	12 (54.54%)	0.41
Mild-moderate	30 (66.67%)	20 (60.60%)	10 (83.33%) ***	0.04
Severe	15 (33.30%)	13 (39.39%)	2 (16.66%)	0.04
Mortality rate	5 (5.2%)	5 (6.75%)	…………..	
Mild-moderate	1 (20.00%) #	1 (1.35%) #	…………..	
Severe	4 (4.16%)	4 (5.40%)	……………	

* *p* < 0.05 as compared to severe COVID-19 male patients on metformin monotherapy, ** *p* < 0.05 relative to severe COVID-19 female patients on metformin monotherapy, *** *p* < 0.05 as compared to severe COVID-19 female patients on metformin plus sitagliptin, # *p* < 0.05 relative to severe COVID-19 male patients.

**Table 7:** Clinical scoring of COVID-19 patients.

Scores	Interpretations
I	Patient has normal activity and does not need hospitalization.
II	Patient has sub-normal activity and does not need hospitalization.
III	Patient needs to be hospitalized without the need for oxygen therapy.
IV	Patient needs to be hospitalized and non-invasive oxygen therapy.
V	Patient needs to be hospitalized and invasive oxygen therapy.
VI	Patient needs to be hospitalized and mechanical ventilation.
VII	Death.

## Data Availability

Data are contained within the article and Supplementary Material.

## Supplementary Materials

The following supporting information can be downloaded at: https://www.mdpi.com/article/10.3390/ph15111361/s1, Table S1. Demographic characteristics of the patients, Table S2. Clinical presentations of Covid-19 patients at time of admission, Table S3. Clinical presentations of Covid-19 patients at time of discharge.
