# Supplementary material for: Potential Therapeutic Benefits of Metformin Alone and in Combination with Sitagliptin in the Management of Type 2 Diabetes Patients with COVID-19
Source: Pharmaceuticals (Basel). 2022 Nov 7;15(11):1361. doi: 10.3390/ph15111361 (PMC9699540; doi:10.3390/ph15111361)
Supplement: Supplementary file 1 [file pharmaceuticals-15-01361-s001.zip › pharmaceuticals-1998609-supplementary.pdf]

Table S1. Demographic characteristics of the patients.

The characteristics	T2DM patients	T2DM patients/Covid-19	P
n	78 (43.57%)	101(56.42%)	0.08
Male sex	50 (64.10%)	79 (78.21%)	0.03*
Age (years)	49.46± 7.97	53.63± 7.99	0.86
Smoking	18 (23.07%)	25 (24.75%)	0.79
Duration of T2DM (years)	5.29± 2.86	5.13±2.94	0.71
**Diabetes therapy**			
Metformin monotherapy	40(51.28%)	55 (54.45%)	0.67
Metformin+ sitagliptin	38 (48.71%)	46 (45.54%)	0.67
**Co-morbidities**			
Obesity	31(39.74%)	44 (43.56%)	0.60
Hypertension	33 (42.30%)	53 (52.47%)	0.17
Dyslipidemia	34(43.58%)	22 (21.78%)	0.001*
Asthma	2 (2.56%)	4 (3.96%)	0.60
Previous stroke	5 (6.41%)	12 (11.88%)	0.21
Ischemic heart disease	8 (10.25%)	10 (9.90%)	0.93
**Concomitant treatments**			
Fenofibrate	29 (37.17%)	11 (10.89%)	0.001*
Statins	4 (5.12%)	4 (3.96%)	0.71
Aspirin	5 (6.41%)	15 (14.85%)	0.07
ACEIs	22 (28.20%)	33 (32.67%)	0.52
ARBs	11(14.10%)	12 (11.88%)	0.66
Montelukast	2 (2.56%)	4 (3.96%)	0.60
Trimetazidine	8 (10.25%)	9 (8.91%)	0.76
Amlodipine	6 (7.69%)	3 (2.97%)	0.15
Prophylactic antibiotics	……..	95 (94.05%)	……..
Antiviral	………	30 (29.70%)	………
Enoxaparin	………	30 (29.70%)	………
Oxygen supplement	………..	30 (29.70%)	………

Data presented as mean ± SD, number (n), percentage (%).*P<0.05, ACEIs: angiotensin converting enzyme inhibitors, ARBs: angiotensin receptor blockers.

Table S2. Clinical presentations of Covid-19 patients at time of admission.

**Clinical presentation**	**Mild-moderate Covid-19 (*n*=71)**	**Severe Covid-19 (*n*=30)**	**P**
**Fever**	62 (87.32%)	29 (96.67%)	0.15
**Mild (<38C)**	59 (95.16%)	1 (3.44%)	0.0001
**severe (>38 C)**	3 (4.83%)	28 (96.56%)	0.0001
**Headache**	44 (61.97%)	27 (90.00%)	0.005
**Dry cough**	55 (67.46%)	29 (96.67%)	0.001
**Dyspnea**	11(15.49%)	30 (100.00%)	0.0001
**Anosmia**	24 (30.80%)	26 (86.67%)	0.0001
**Sweating**	17 (23.94%)	29 (96.67%)	0.0001
**Vomiting/diarrhea**	12 (16.90%)	23 (76.67%)	0.0001
**Abdominal pain**	10 (14.08%)	22 (73.30%)	0.0001
**Hypoxemia**	……..	30 (100.00%)	……..
**Myalgia**	26 (36.61%)	30 (100.00%)	0.0001
**Asthenia**	29 (40.84%)	30 (100.00%)	0.0001

Table S3. Clinical presentations of Covid-19 patients at time of discharge

Clinical presentation	Mild-moderate Covid-19 (*n*=70)	Severe Covid-19 (*n*=26)	Difference (%)	P
Mortality rate	1(1.40%)	4(13.13%)	12.99	0.002
Mild fever	2 (2.85%)	11 (42.30%)	39.45	0.00001
Headache	30 (42.85%)	20 (76.92%)	39.41	0.003
Dry cough	3 (4.28%)	13 (50.00%)	72.64	0.0001
Dyspnea	1(0.14%)	10 (38.46%)	38.32	0.0001
Anosmia	32(45.71%)	20 (76.92%)	31.21	0.006
Sweating	………	2 (7.69%)		……..
Vomiting/diarrhea	………	2 (7.69%)		……..
Abdominal pain	………	1 (3.84%)		……..
Hypoxemia	………	10 (38.46%)		……..
Myalgia	……..	2 (7.69%)		……..
Asthenia	………	4(13.13%)		……..
